# Supplementary material for: Age-Related Changes in Survival Behaviour in Parasite-Free Hatchery-Reared Rainbow Trout (Oncorhynchus mykiss)
Source: Animals (Basel). 2024 Apr 27;14(9):1315. doi: 10.3390/ani14091315 (PMC11083660; doi:10.3390/ani14091315)
Supplement: Supplementary file 1 [file animals-14-01315-s001.zip › animals-2948808-supplementary.pdf]

Supplementary material:

Figure S1: Image of the plus maze behavioural tank set up for the habitat choice test.

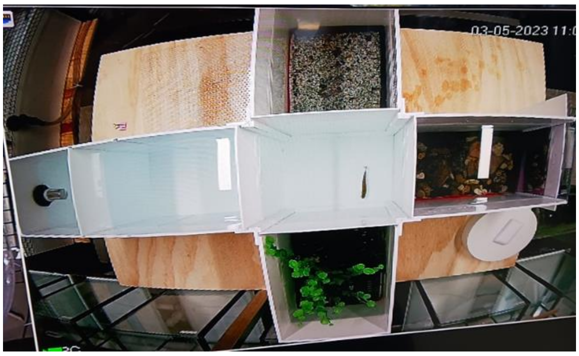

Figure S2: Photo of Nankeen Night Heron (*Nycticorax caledonicus*) used for the avian predator avoidance test

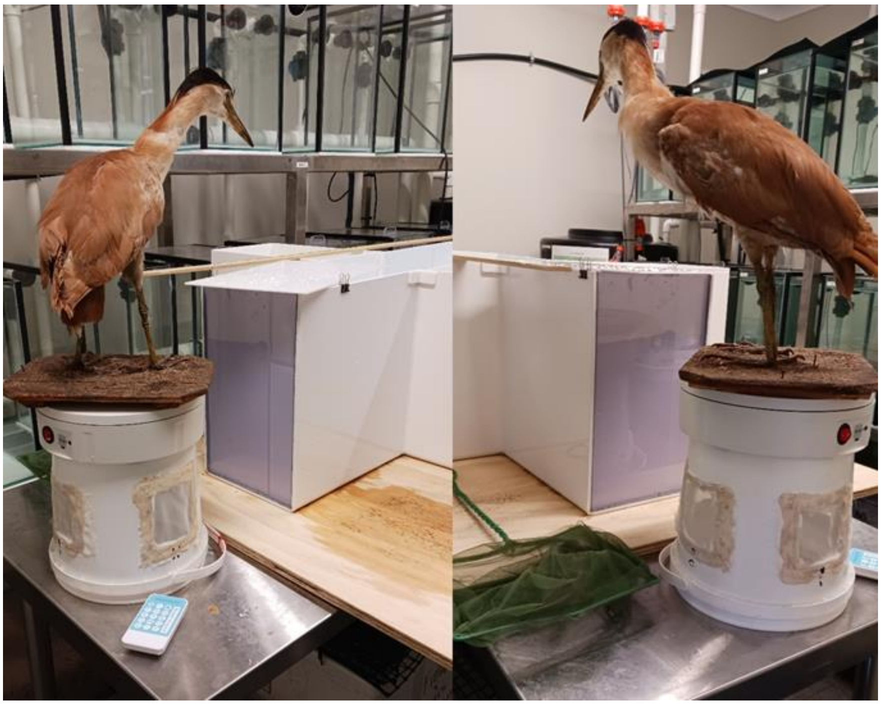

Figure S3 Diagram of tank used for the fish predator avoidance test.

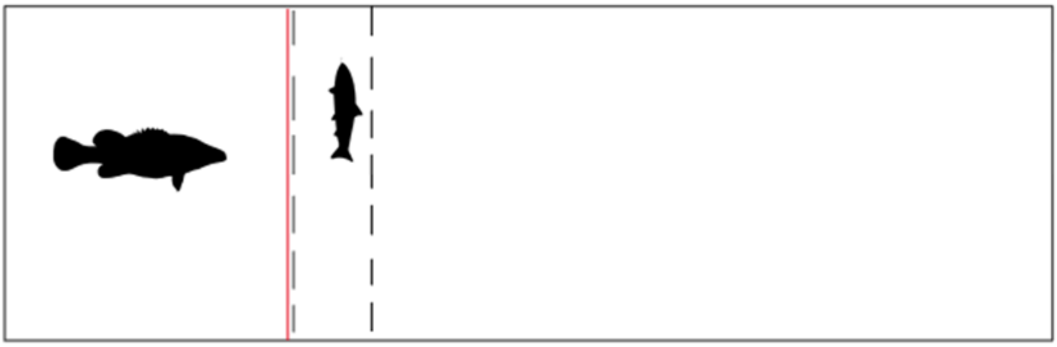

Figure S4 – Median and spread boxplots of latency of emergence in trout of six ages.

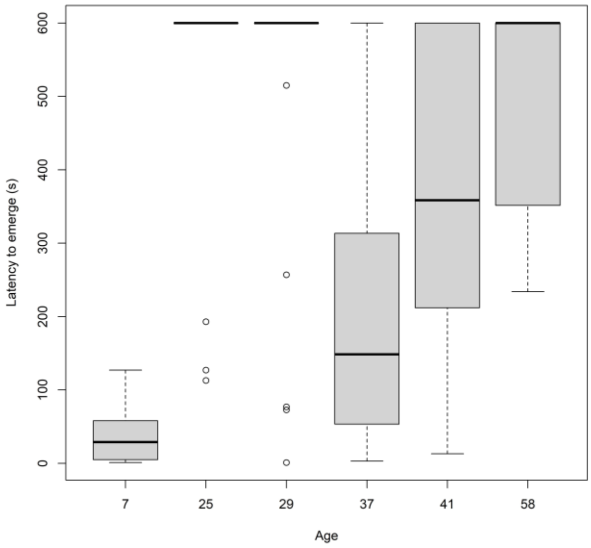

Figure S5: Percentage of trout biting the bait in one hour at six ages.

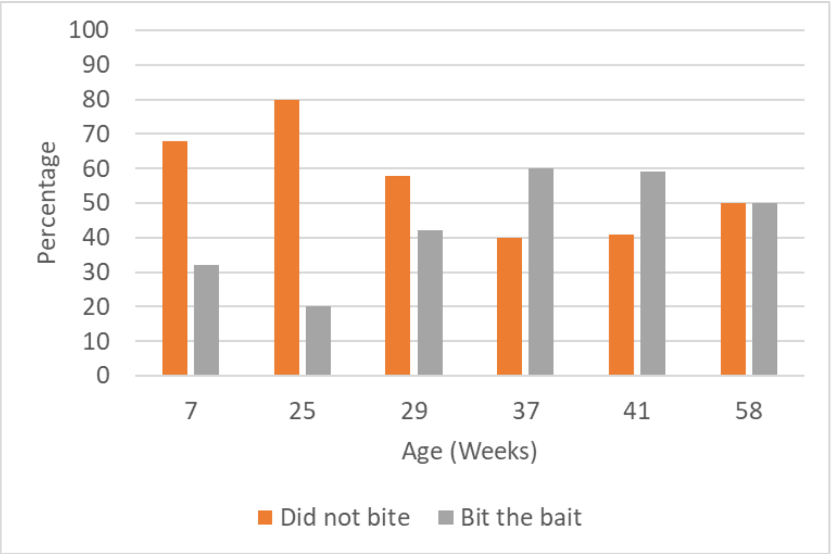

Figure S6: Top photo: Gill filaments. Middle photo: Liver. Bottom photo, clockwise from top: Gill filaments and operculum of 29-week-old trout; Jaw, heart, eyes, spleen and liver of 29-week-old trout (left to right); gastrointestinal tract of 29-week-old trout; brain of 29-week-old trout and fillets of body wall, spine and kidney of 29-week-old trout.

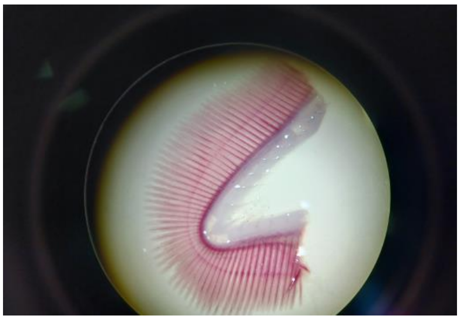

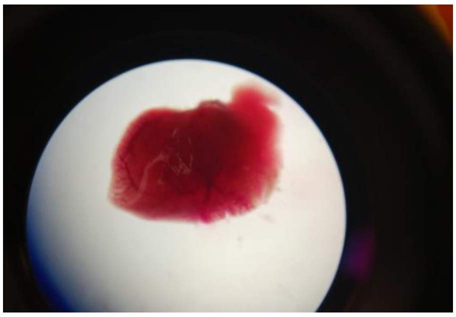

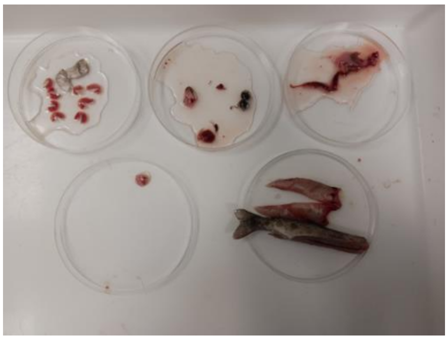

Table S1: Summary of behavioural tests

Test	Acclimatisation/start	Apparatus	Measures recorded
**Emergence**	Fish confined to "refuge" area for 10 minutes. Guillotine door then lifted allowing access to plus maze through 10x60cm opening.	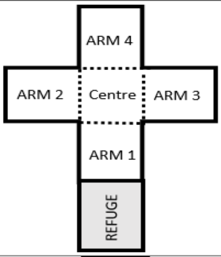	Latency (seconds) for the whole body to emerge from the refuge.
**Exploration**	Immediately after the emergence test. Fish were prevented from returning to the Refuge area by a guillotine door.	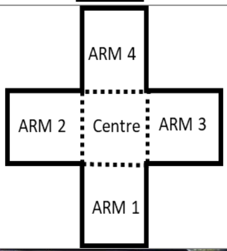	Latency to cross into the centre and number of arms entered.
**Habitat choice**	Fish confined to "centre" area to acclimatise and clear doors (hashed lines) were then removed. Different substrate in each arm: 1) pebbles, 2) vegetation, 3) small rocks and 4) empty (order and position randomised).	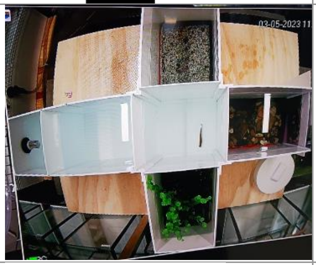	Number of entries into each arm and total time spent in each arm.
**Response to bird predator**	Fish confined to an area beside a model night heron for 10 minutes. The trout is then exposed to the bird visible through the clear glass and at the same time the guillotine door (red line) is removed allowing the fish to move away from the predator.	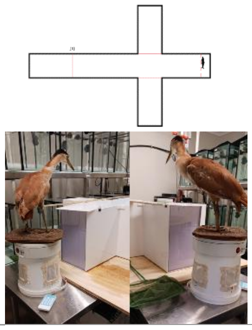	Type of response (avoid or freeze), latency to move away from the predator and number of startle responses.
**Response to Fish predator**	Fish confined to an area beside a predator (large Murray cod) for 10 minutes. A visual barrier is then removed allowing the trout to see the predator through a clear perforate glass and at the same time the guillotine door (dashed line) is removed allowing the fish to move away from the predator.	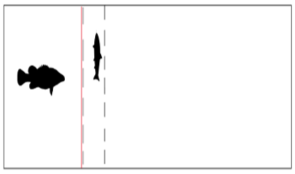	Type of response (avoid or freeze), latency to move away from the predator and number of startle responses.
**Novel food (neophobia test)**	In home tanks, fish is presented with a gulp bait presented on a barbless, blunt hook for one hour.	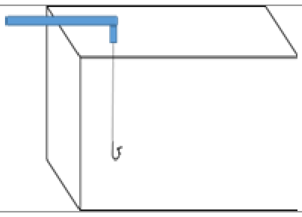	The line is attached to a force displacement transducer to record time and force of the first pull on the bait.

Table S2: Z values and adjusted P values (in brackets) of number of arms entered in the exploration test. Post-hoc pairwise comparisons using Dunn's test.

**Age**	**7**	**25**	**29**	**37**	**41**	**58**
**7**		6.6 (<0.0001)	6.8 (<0.0001)	2.7 (0.06)	4.9 (<0.0001)	5.2 (<0.0001)
**25**			0.1 (0.9)	3.8 (0.002)	1.9 (0.3)	0.4 (1.0)
**29**				3.8 (0.002)	1.9 (0.3)	0.3 (0.9)
**37**					2.0 (0.3)	2.8 (0.04)
